# Detection of liver cirrhosis in standard T2-weighted MRI using deep transfer learning

**DOI:** 10.1007/s00330-021-07858-1

**Published:** 2021-05-11

**Authors:** Sebastian Nowak, Narine Mesropyan, Anton Faron, Wolfgang Block, Martin Reuter, Ulrike I. Attenberger, Julian A. Luetkens, Alois M. Sprinkart

**Affiliations:** 1 Department of Diagnostic and Interventional Radiology, Quantitative Imaging Lab Bonn (QILaB), University Hospital Bonn (Universitätsklinikum Bonn), Venusberg-Campus 1, 53127 Bonn, Germany; 2 Image Analysis, German Center for Neurodegenerative Diseases (DZNE), Bonn, Germany; 3 A.A. Martinos Center for Biomedical Imaging, Massachusetts General Hospital, Boston, MA USA; 4 Department of Radiology, Harvard Medical School, Boston, MA USA

**Keywords:** Deep learning, Neural networks, computer, Magnetic resonance imaging, Liver cirrhosis

## Abstract

**Objectives:**

To investigate the diagnostic performance of deep transfer learning (DTL) to detect liver cirrhosis from clinical MRI.

**Methods:**

The dataset for this retrospective analysis consisted of 713 (343 female) patients who underwent liver MRI between 2017 and 2019. In total, 553 of these subjects had a confirmed diagnosis of liver cirrhosis, while the remainder had no history of liver disease. T2-weighted MRI slices at the level of the caudate lobe were manually exported for DTL analysis. Data were randomly split into training, validation, and test sets (70%/15%/15%). A ResNet50 convolutional neural network (CNN) pre-trained on the ImageNet archive was used for cirrhosis detection with and without upstream liver segmentation. Classification performance for detection of liver cirrhosis was compared to two radiologists with different levels of experience (4^th^-year resident, board-certified radiologist). Segmentation was performed using a U-Net architecture built on a pre-trained ResNet34 encoder. Differences in classification accuracy were assessed by the *χ*^*2*^-test.

**Results:**

Dice coefficients for automatic segmentation were above 0.98 for both validation and test data. The classification accuracy of liver cirrhosis on validation (vACC) and test (tACC) data for the DTL pipeline with upstream liver segmentation (vACC = 0.99, tACC = 0.96) was significantly higher compared to the resident (vACC = 0.88, *p* < 0.01; tACC = 0.91, *p* = 0.01) and to the board-certified radiologist (vACC = 0.96, *p* < 0.01; tACC = 0.90, *p* < 0.01).

**Conclusion:**

This proof-of-principle study demonstrates the potential of DTL for detecting cirrhosis based on standard T2-weighted MRI. The presented method for image-based diagnosis of liver cirrhosis demonstrated expert-level classification accuracy.

**Key Points:**

*• A pipeline consisting of two convolutional neural networks (CNNs) pre-trained on an extensive natural image database (ImageNet archive) enables detection of liver cirrhosis on standard T2-weighted MRI.*

*• High classification accuracy can be achieved even without altering the pre-trained parameters of the convolutional neural networks.*

*• Other abdominal structures apart from the liver were relevant for detection when the network was trained on unsegmented images.*

**Supplementary Information:**

The online version contains supplementary material available at 10.1007/s00330-021-07858-1.

## Introduction

Liver cirrhosis is the end stage of chronic liver disease and a major global health condition, especially due to its variety of severe complications caused by portal hypertension such as variceal bleeding, ascites, and hepatic encephalopathy [1]. Although liver biopsy is the gold standard for the detection of cirrhosis, imaging has a particularly important role in the evaluation of the disease [2]. Imaging is primarily used to characterize the morphologic manifestations of cirrhosis, evaluate the presence and the effects of portal hypertension, and screen for hepatocellular carcinoma. However, morphologic characteristics of cirrhosis are often detected incidentally in patients with unsuspected cirrhosis. It is therefore not unusual that radiologists presume an initial diagnosis of cirrhosis [3].

To assume a diagnosis of liver cirrhosis, different morphological criteria have been described for standard imaging modalities [2]. However, most of these findings are subjective, susceptible to inter-observer variability, and often lack high overall accuracy for the detection of cirrhosis [4]. Therefore, quantitative analyses, which could improve the objectivity and reading performance in the identification of liver cirrhosis, are of great interest [5].

A method that could objectively assess relevant features automatically within radiological images could support the radiologist in diagnosing liver cirrhosis, leading to greater accuracy and less variation in reading performance. Since 2012, when a deep learning technique has shown superior performance in the prominent ImageNet challenge for the first time, especially CNNs have become the gold standard for image classification and segmentation [6]. Deep learning methods have been continuously improved and successfully applied in various disciplines, including medical imaging [7–12].

However, a disadvantage of CNNs is the requirement of a large number of pre-classified images, which serve as training data. Instead of training a neural network from scratch with a small data set, it has proven advantageous to use a technique called transfer learning [13]. The basic idea is to use a CNN pre-trained e.g. on a large natural image dataset, which has already been trained to recognize complex patterns and then adapt it to a different task. This technique has recently been successfully applied to a variety of segmentations and classification problems of medical image data [14–16].

The aim of this study was to investigate the capabilities of deep transfer learning (DTL) to identify liver cirrhosis in standard T2-weighted MRI and to evaluate the diagnostic performance against radiologists with different levels of experience.

## Materials and methods

**Fig. 1:**
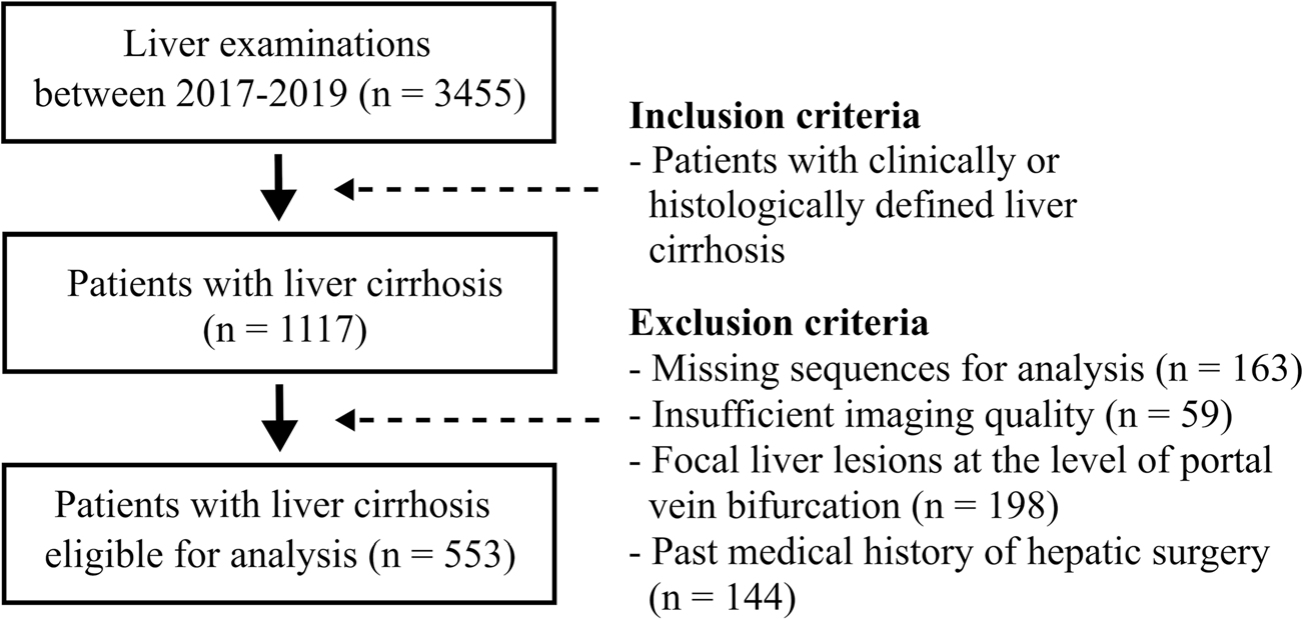
Flowchart illustrating the inclusion and exclusion criteria for the group of patients with liver cirrhosis for this study

This retrospective study was approved by the institutional review board with a waiver of written informed consent. Patients who underwent liver MRI at our institution for standard diagnostic purposes between 2017 and 2019 were included. Two groups of patients were identified and included in the final study cohort:

i. Patients with known liver cirrhosis of any stage: Inclusion criterion was the presence of histologically or clinically defined liver cirrhosis of any clinical disease severity. Exclusion criteria were the presence of focal liver lesions at the level of portal vein bifurcation or a past medical history of hepatic surgery (Fig. 1).
ii. Patients without known liver disease: From the same period, a randomly selected control group was recruited, which consisted of patients without known liver disease. Exclusion criteria for the control group were the same as those applied for the cirrhosis group.

Patient characteristics were retrieved from the clinical information management system of the referring institution. An overview of the MRI indications for the two groups is provided in Supplement S1.

As this study aimed to determine the diagnostic utility of DTL to detect liver cirrhosis based on morphological hallmarks of liver cirrhosis, T2-weighted imaging was used for analysis. In detail, images of a standard T2-weighted respiratory triggered multi-slice turbo spin echo sequence with non-Cartesian k-space filling with radial rectangular blades (Multi Vane XD) were used. For each patient, a single-slice image at the level of the caudate lobe was exported for DTL analysis (N.M. with 1 year of experience in the field of clinical abdominal imaging). All examinations were performed on clinical whole-body MRI systems (Philips, Ingenia 1.5 T and 3 T). Detailed imaging parameters are listed in Supplement S2.

Image data were randomly divided into training data (70%), validation data (15%), and test data (15%) using a custom Matlab script (MathWorks). Details of the preprocessing prior to training are listed in Supplement S3.

Images were analyzed using two different processing pipelines. In the first pipeline, an image segmentation network was applied prior to the classification task. In the second pipeline, the classification was performed directly on the unsegmented images.

For segmentation, a CNN following the principle architecture of a U-net model was implemented [17]. Its descending encoder part is identical to a CNN with residual connections known as ResNet34 that was pre-trained on the ImageNet database [18]. The ground truth for the training of the segmentation CNN was generated by a radiology resident (N.M.) by manually delineating the liver using in-house tools developed in Matlab and verified by a board-certified radiologist (J.A.L.).

**Fig. 2:**
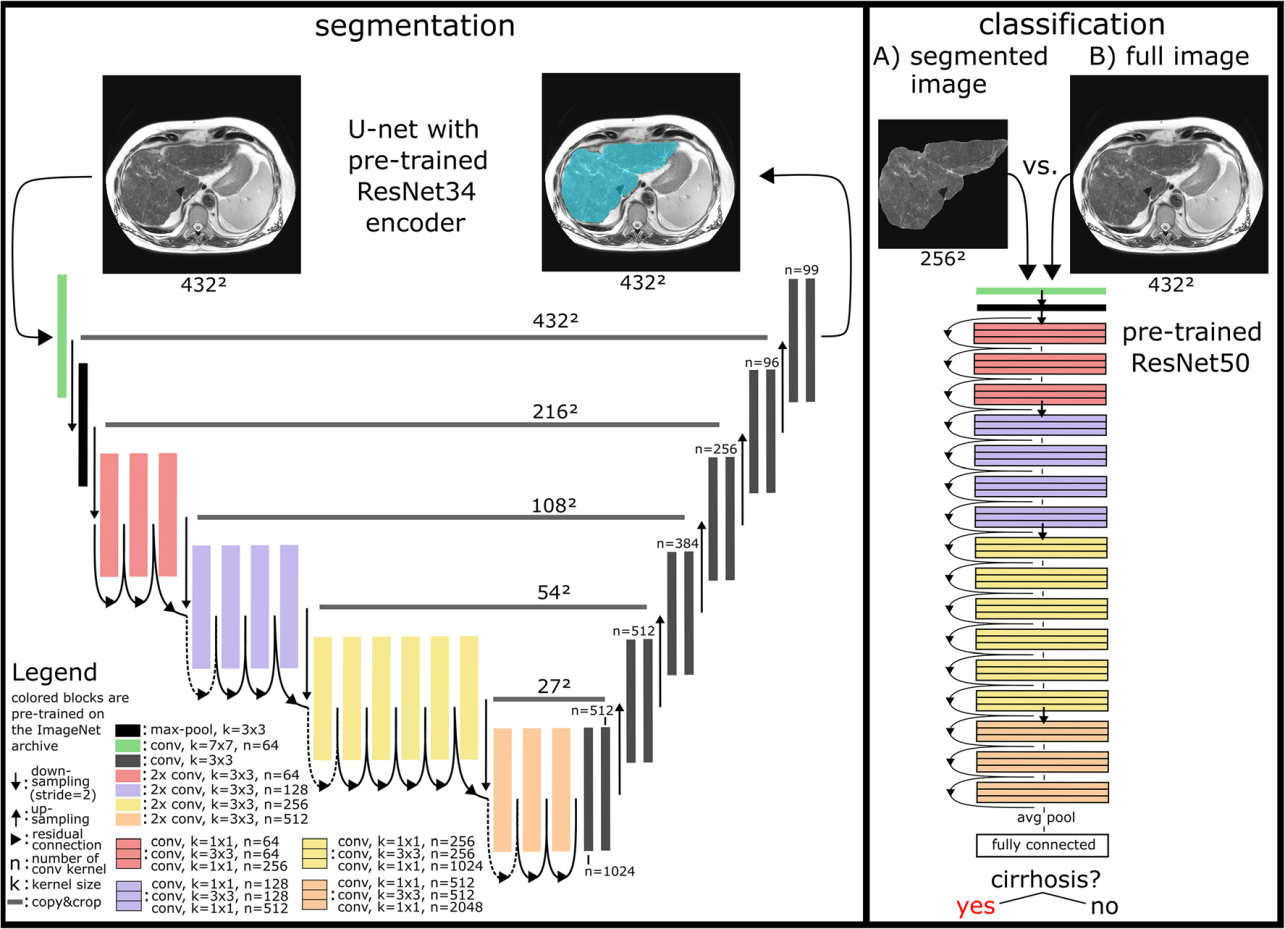
Details of the presented deep transfer learning (DTL) pipeline for detection of liver cirrhosis. The segmentation network (left) is based on a U-net architecture, with a ResNet34 convolutional neural network (CNN) as encoder, pre-trained on the ImageNet archive. For the classification task (right), a pre-trained ResNet50 CNN was employed. The classification performance of the DTL pipeline including liver segmentation (**A**) was compared to a classification based on the original, unsegmented images (**B**)

ResNet50 as a well-established CNN with 50 trainable layers and residual connections was used for the classification task in both pipelines. The model was pre-trained on the ImageNet archive and implemented in pytorch’s torchvision package [19]. Detailed descriptions of the segmentation and classification CNN architectures can be found in Fig. 2 and Supplement S4.

The DTL methods developed in this work were trained in two phases. First, only non-pretrained layers were trained and all pre-trained parameters of the convolutional layers were kept constant. To further investigate whether varying the pre-trained parameters may improve the reading performance of the CNN, the parameters of the pre-trained convolutional layers were made variable in a second phase. The one cycle learning rate policy was applied for fine-tuning of the pre-trained models for liver segmentation and classification of liver cirrhosis [20]. All experiments and evaluations were performed with python and fastai, a deep learning application programming interface for pytorch [21]. Further details of the experimental design and the hyper-parameters used for training are given in Supplement S5.

To compare the performance of the DTL analyses to the performance of healthcare professionals at different experience levels, validation and test data were also classified independently by a radiology resident (A.F.) with 4 years of experience in abdominal imaging and a board-certified radiologist (J.A.L.) with 8 years of experience in abdominal imaging.

The 95% confidence interval of the DTL-based classification accuracy was determined by the Clopper-Pearson method and a *χ*^2^-test was performed to test for significant differences in accuracy between the DTL-based classification and the readers in SPSS Statistics 24 (IBM). For the test set, calculations of balanced accuracy, receiver operating characteristic, and precision-recall analyses were performed with scikit-learn 0.23.2 [22–24].

In order to assess the classification performance of the entire first pipeline (including prior segmentation), the segmentations of the CNN (instead of manual segmentations) were used for the validation and test set of the classification network. In addition to evaluating the method by its performance on the validation and test data set, gradient-weighted class activation maps (Grad-CAMs) were generated [25]. This technique is proposed to add visual information to radiological images, describing areas of the image that affect the prediction of the CNN [26]. These colored prediction maps were visually inspected and the image areas contributing to the CNN’s prediction of cirrhosis were quantified separately for both patient groups.

## Results

A total of 713 patients (342 female, mean age: 58 ± 14 years) were included. Of those, examinations of 572 patients were acquired at a field strength of 1.5 T. The remainder were examined on 3.0 T. A total of 553 patients (248 female, mean age: 60 ± 12 years) with a confirmed diagnosis of liver cirrhosis based on clinical or histopathological criteria were included (Fig. 1). The control group consisted of 160 subjects (94 female, mean age: 49 ± 18 years) without history of liver disease. A training set with 505 subjects (244 female, mean age: 58 ± 14 years), a validation set with 104 subjects (49 female, mean age: 57 ± 14 years), and a test set with 104 subjects (49 female, mean age: 58 ± 15 years) were compiled by random selection, while maintaining the proportion of control patients to patients with cirrhosis. The DTL method for segmentation of the liver in the transverse T2-weighted MRI images developed on the training set showed Dice values of 0.984 for the validation set and 0.983 for the test set.

In the subsequent training of the classification network ResNet50 for the identification of cirrhosis based on segmented images, an accuracy (ACC) of 0.99 (95% confidence interval: 0.95–1.00) for validation data (vACC) and 0.96 (0.90–0.99) for test data (tACC) was achieved. For the classification on unsegmented images, vACC was 0.97 (0.92–0.99) and tACC was 0.95 (0.89–0.98). The accuracy of the DTL pipeline for classification of cirrhosis with prior segmentation of the organ was significantly higher compared to the resident (vACC = 0.88, *p* < 0.01; tACC = 0.91, *p* = 0.01) as well as the board-certified radiologist (vACC = 0.96, *p* < 0.01; tACC = 0.90, *p* < 0.01) (Table 1). Modifications of pre-trained parameters did not improve segmentation and classification accuracy significantly (Table 2). On the test set, a balanced accuracy value of 0.90 was observed for the DTL method based on unsegmented images. Balanced accuracy values of 0.92 were observed for the DTL method based on segmented images, as well as for the radiology resident and board-certified radiologist. For the DTL method, the balanced accuracy of 0.92 is derived from a sensitivity of 1, which was higher than that of the radiology resident and board-certified radiologist (0.91, 0.89) and a specificity of 0.83, which was lower than that of the radiology resident and board-certified radiologist (0.92, 0.96).

**Table 1:** Accuracy (ACC), balanced accuracy (BACC), sensitivity (Sens)**,** and specificity (Spec) for identification of liver cirrhosis for validation (vACC, vBACC, vSens, vSpec) and test (tACC, tBACC, tSens, tSpec) of the deep transfer learning (DTL) method based on unsegmented images and based on images with prior segmentation of the liver. The accuracy of the DTL approaches was also compared to a radiological resident and a board-certified radiologist. Statistical difference was assessed by *χ*^**2**^-test

Reader/method	vACC	*p* value (vAcc)	tACC	*p* value (tAcc)	vBACC	tBACC	vSens	tSens	vSpec	tSpec
ResNet50 (segmented liver)	0.99	-	0.96	-	0.99	0.92	0.99	1	1	0.83
ResNet50 (full image)	0.97	*p* = 0.04	0.95	*p* = 0.61	0.97	0.90	0.98	1	0.96	0.79
Board-certified radiologist	0.96	*p* < 0.01	0.90	*p* < 0.01	0.98	0.92	0.95	0.89	1	0.96
Radiology resident (4th year)	0.88	*p* < 0.01	0.91	*p* = 0.01	0.93	0.92	0.85	0.91	1	0.92

**Table 2:** Dice values of the segmentation convolutional neural network (CNN) and classification accuracy of liver cirrhosis of the classification CNN at different stages of the training experiments. In the first stage of training the segmentation CNN, a Dice score of 0.9828 was achieved by optimizing the convolutional layers of the random-initialized decoder and remaining the parameters of the pre-trained ResNet34 encoder unchanged. In the following three stages that started from the model state of the previous stage, only minor improvements of 0.001 of the Dice score were achieved. In these stages, the convolutional layers of the pre-trained ResNet34 encoder were made variable, whereby the learning rate (LR) increased linearly from the first to the last layer of the CNN. In the first stage of training the classification CNN, an accuracy of 0.99 for the segmented images and 0.97 for the unsegmented images were achieved by optimizing the output layer of the ResNet50 CNN only. The following stages that started from the best previous model state did not lead to an improvement in accuracy and showed only minor improvements of the cross-entropy loss. Also in the last three stages, where the convolutional layers of the pre-trained ResNet50 were made variable with learning rates increased linearly from the first to the last layer of the CNN, no improvement in accuracy could be observed. Detailed descriptions of the training experiments can be found in Supplement S5

	Training stage	Epochs	Max LR last layer decoder	Max LR first layer encoder	Dice on validation set	
Segmentation network (U-net like with ResNet34 encoder)	1	80	0.001	Frozen	0.9828	
	2	40	0.0005	0.000005	No improvement	
	3	40	0.0005	0.00005	0.9837	
	4	40	0.0005	0.0005	0.9838	
	Training stage	Epochs	Max LR output layer	Max LR first layer	Accuracy and cross-entropy loss (segmented image)	Accuracy and cross-entropy loss (full image)
Classification network (ResNet50)	1	80	0.1	Frozen	0.99, 0.1452	0.97, 0.325
	2	40	0.01	Frozen	No improvement	0.97, 0.2151
	3	40	0.001	Frozen	No improvement	No improvement
	4	40	0.0001	0.000001	No improvement	0.97, 0.2025
	5	40	0.0001	0.00001	0.99, 0.1450	No improvement
	6	40	0.0001	0.0001	0.99, 0.1339	No improvement

**Fig. 3:**
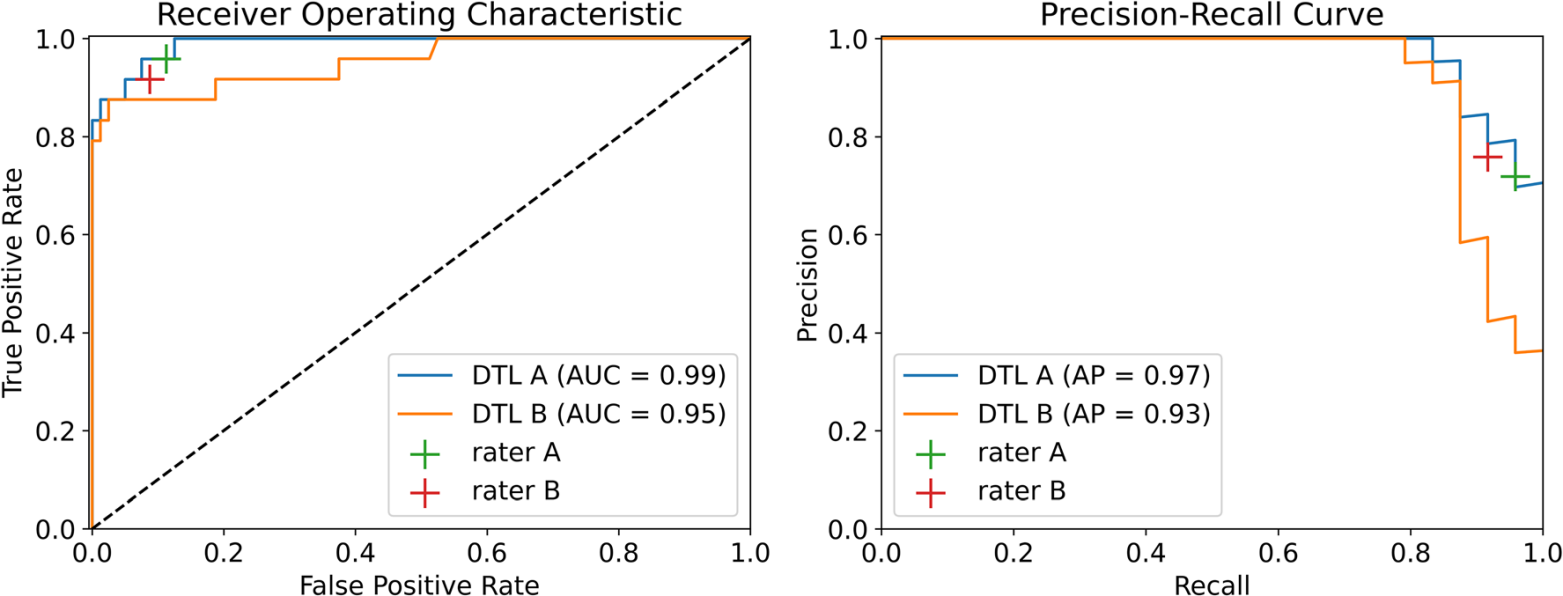
Liver cirrhosis classification performance of the deep transfer learning (DTL) methods trained on the segmented images (DTL A) or unsegmented images (DTL B) and of the radiology resident (rater A) and the board-certified radiologist (rater B) on the test set, illustrated by receiver operating characteristic and precision-recall curves and area under the curve (AUC) and average precision (AP) values

Receiver operating characteristic and precision-recall curves for the test data set are shown in Fig. 3. For the DTL method trained on segmented images, an area under the curve (AUC) of 0.99 and an average precision (AP) of 0.97 and for the DTL method trained on the unsegmented images, an AUC of 0.95, and an AP of 0.93 were determined.

**Fig. 4:**
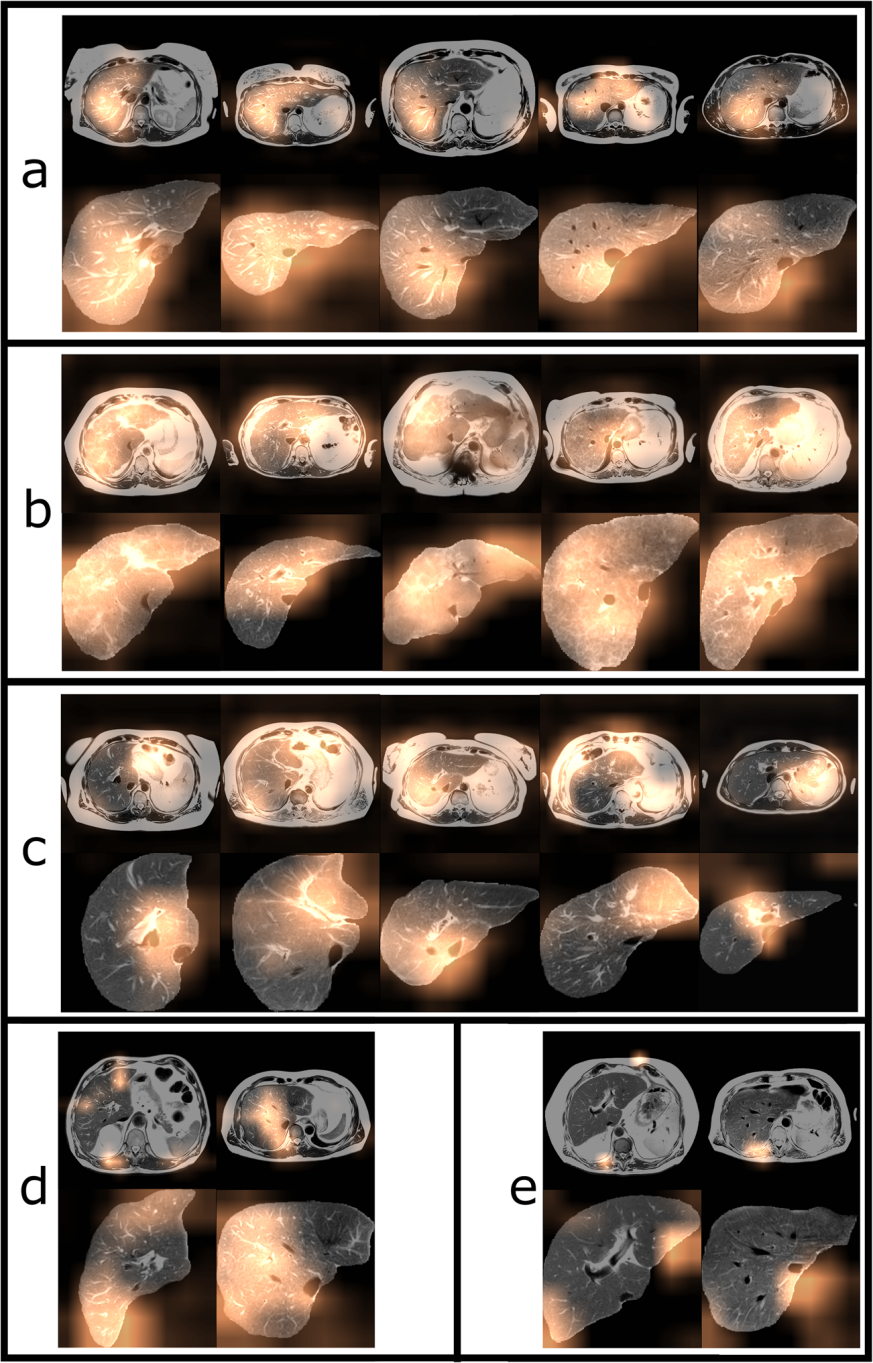
Gradient-weighted class activation maps for unsegmented and segmented images from the test set. The overlays highlight regions that had high impact on classification in patients without cirrhosis (**a**) and patients with cirrhosis (**b**). Patients with and without cirrhosis that were correctly classified by the DTL methods but incorrectly classified by the certified radiologist are shown in **c**. Examples of images with a disagreeing classification of the two DTL methods, where the image was only correctly classified with prior liver segmentation are shown in **d**. Images that were misclassified by both DTL methods, but correctly classified by the certified radiologist are shown in **e**

Figure 4 shows exemplary images from the test set with colored maps indicating areas which were particularly relevant for the decision of the classifier. The results of the visual inspection are presented in Table 3. In the first pipeline with upstream segmentation, the caudate lobe was highlighted in 47.5% of the images classified as cirrhosis and in 25% of the images classified as no cirrhosis. In every fifth (20.8%) of the segmented images classified as no cirrhosis, the transition zone of the caudate lobe to the image background was highlighted.

**Table 3:** Evaluation of the gradient-weighted class activation maps of the test set. The maps of the predictions of the deep transfer learning method, trained on segmented images and images without liver segmentation, were visually inspected and it was recorded which image areas were highlighted, separately for both patient groups. Note that several areas of the image were highlighted, so the percentages of the different image areas do not add up to 100% within a patient group. The liver areas were divided into left, right hepatic, and caudate lobe. For the segmented images, it was also noted whether image areas at the transition zone of the caudate lobe to the image background were highlighted. For the full images, highlighted areas near the stomach, spleen, gastroesophageal junction, and spinal muscles were observed

Unsegmented images	Patient group	Right hepatic	Left hepatic	Caudate lobe	Spleen	Stomach	Gastroesophageal junction	Spinal musculature
	Cirrhosis	53.8%	35%	22.5%	6.3%	22.5%	12.5%	2.5%
	No cirrhosis	83.3%	16.7%	0	0	8.3%	0	29.2%
Segmented images	Patient group	Right hepatic	Left hepatic	Caudate lobe	Border caudate lobe/background		-	-
	Cirrhosis	53.8%	28.8%	47.5%	2.5%		-	-
	No cirrhosis	58.3%	20.8%	25%	20.8%		-	-

In the second pipeline, based on unsegmented images, additional highlighted areas outside of the liver were identified. In images classified as cirrhosis, the spleen area was highlighted in 6%, the stomach area in 22.5%, and the gastroesophageal junction in 12.5%. In 29.2% of the CNN’s negative predictions, spinal musculature was highlighted.

## Discussion

This proof-of-principle study demonstrates the feasibility of automatic detection of liver cirrhosis by DTL based on a standard T2-weighted MRI. The deep learning approach with prior segmentation of the liver provides classification accuracy at expert level.

To date, no other work has investigated the use of a DTL approach for the detection of liver cirrhosis in standard T2-weighted MRI sequences. There are recent studies based on gadoxetic acid–enhanced MRI imaging that classifies fibrotic pathologies of the liver by methods of deep learning and radiomics [27, 28]. However, these methods are trained from scratch and they require a manual definition of region of interests. In contrast to that, the method proposed in the current study does not require manual segmentation since the liver is segmented automatically with high precision.

Recent studies based on ultrasound imaging also used DTL methods pre-trained on the ImageNet archive [29, 30]. Of note, in both mentioned studies, the pre-trained parameters were not kept constant during training. Particularly the first few layers of the pre-trained CNNs have learned to recognize very general image features such as edges and shapes during the training with the ImageNet data set [31]. The ability to extract these features is a benefit of transfer learning, and therefore, other groups proposed to first optimize only the output layer of the network prior to changing the pre-trained parameters of the CNN [15, 32].

In order to examine whether altering the pre-trained parameters of the DTL methods is beneficial for the identification of cirrhosis, the CNNs were trained in two phases in this work, with frozen and unfrozen pre-trained parameters. Interestingly, the accuracy on the validation data set of both methods did not further increase by unfreezing the pre-trained parameters. Hence, the learned feature extraction capability from the training on the natural image data set of e.g. cars, animals, and buildings was generalized to identify liver cirrhosis on an expert level in standard T2-weighted MRI.

A further aim of our study was to investigate, whether prior segmentation of the liver is beneficial for this classification task. Interestingly, both variants (with and without prior segmentation) achieved high accuracy. However, the accuracy for the detection of liver cirrhosis was slightly higher for the DTL pipeline with prior segmentation. This result may be attributed to the following advantages of upstream segmentation:

i. The network is forced to focus on the area, where pathological alterations are primarily expected.
ii. Image areas that are not in focus of the analysis are prevented to have an impact on the normalization step [33].
iii. Using only the image areas of the organ allows to train the classification model with smaller image matrices and thus larger batch size, which is considered beneficial for the applied learning rate policy [20].

For both methods, image areas relevant for the CNN’s decision were investigated applying the Grad-CAM method [25]. The results indicate that the caudate lobe area is important for the DTL methods for the detection of liver cirrhosis trained on either segmented or unsegmented images. Interestingly, the Grad-CAM evaluations of the DTL method based on the unsegmented images showed that in some cases, image areas outside of the liver were relevant. This indicates that the CNN might also base the prediction of cirrhosis on accompanying signs of cirrhosis, such as spleen hypertrophy, venous alterations like fundus varices, or the general vital status of the patient according to muscle structure. This observation motivates further studies to investigate if deep learning methods may also reliably detect accompanying effects of cirrhosis.

Future work should also address whether a multi-task-learning architecture, which would simultaneously optimize segmentation and classification performance, has advantages over the presented pipeline. In addition, the method could be extended by an automated selection of the 2D slice at the level of the caudate lobe to allow fully automated prediction of cirrhosis based on T2-weighted imaging.

Our study has several limitations. First, the DTL model has been trained for the identification of liver cirrhosis only and does not support the detection of very early signs of tissue fibrosis, which might be present in early hepatopathy. However, this was not the aim of this proof-of-principle study, but to investigate the hypothesis that ImageNet pre-trained models are generalizable to T2-weighted MRI imaging and allow the assessment of imaging features of liver cirrhosis. The investigation of an automated classification of early signs of tissue fibrosis and different stages of fibrosis will be the next step in the evaluation of deep transfer learning–based approaches based on standard T2-weighted MRI imaging.

Our study collective included a broad range of cirrhosis severities (according to the Child-Pugh score) and different etiologies of cirrhosis. To account for the difference in the number of patients with liver cirrhosis and patients without liver disease, additional performance measures were assessed. According to the balanced accuracy, the method trained on segmented images performs at expert level. However, the DTL method shows a higher sensitivity and a lower specificity compared to the board-certified radiologist, which may be a result of the class imbalance of the dataset. An expert level classification performance of the DTL method trained on segmented images is furthermore underlined by the precision-recall analysis.

Another limitation is that the classification was based solely on T2-weighted images. In contrast to that, additional pieces of information such as different MRI sequences as well as clinical and laboratory parameters are typically available for diagnosis in clinical routine. However, in our study, high diagnostic accuracy was shown for both the classifier and clinical experts, even if the diagnosis was based on only one anatomical sequence. Future studies may evaluate whether a multi-parametric approach or the inclusion of clinical parameters can further improve diagnostic performance.

## Conclusion

This proof-of-principle study demonstrates the potential of DTL for the detection of cirrhosis based on standard T2-weighted MRI. The DTL pipeline for the image-based diagnosis of liver cirrhosis demonstrated classification accuracy at expert level. An application of the pipeline could support radiologists in the diagnosis of liver cirrhosis and has the potential to improve consistency of reading performance.

## Supplementary information

ESM 1 (DOCX 42 kb)

## Abbreviations

ACC: Accuracy
AP: Average precision
AUC: Area under the curve
CNN: Convolutional neural network
DTL: Deep transfer learning
