# Supplementary material for: Detection of liver cirrhosis in standard T2-weighted MRI using deep transfer learning
Source: Eur Radiol. 2021 May 11;31(11):8807–15. doi: 10.1007/s00330-021-07858-1 (PMC8523404; doi:10.1007/s00330-021-07858-1)
Supplement: Supplementary file 1 — (DOCX 42 kb) [file 330_2021_7858_MOESM1_ESM.docx]

**SUPPLEMENTARY MATERIALS**

**S1 Main MRI indications**

| **Main MRI indications** | **Patients with liver cirrhosis n=553 (%)** | **Control group n=160 (%)** |
| --- | --- | --- |
| **Hepatocellular carcinoma (HCC) screening** | 259 (46.8) | - |
| **Evaluation for liver transplantation** | 20 (3.6) | - |
| **Treatment monitoring** | 76 (13.7) | - |
| **Evaluation for transjugular intrahepatic portosystemic shunt (TIPS) implantation** | 64 (11.6) | - |
| **Exclusion of hepatic / abdominal masses** | - | 56 (35) |
| **Focal lesion evaluation** | 95 (17.2) | 70 (43.8) |
| **Follow up of known lesions** | 26 (4.7) | 11 (6.8) |
| **Other** | 13 (2.4) | 23 (14.4) |

**Table S1**: MRI indications of the examinations of the study cohort.

**S2 Scanner and Imaging details**

Images were acquired at three MR systems. Scanner 1: Philips Ingenia, field strength = 3 T, maximum gradient amplitude= 80 mT/m, maximum slew rate = 200 T/m/s. Scanner 2 and 3: Philips Ingenia, field strength = 1.5 T, maximum gradient amplitude = 45 mT/m, maximum slew rate = 200 T/m/s. Table S2 shows the range as well as mean and median of basic sequence parameters of the T2-weighted MRI sequences grouped by training, validation and test dataset. Typical sequence parameters at 1.5 T were: echo time (TE) = 80 ms, repetition time (TR) = 1800 ms, flip angle = 90°, slice thickness = 5 mm, pixel spacing = 0.88 mm. Typical sequence parameters at 3 T were: TE = 80 ms, TR =2200 ms, flip angle = 90°, slice thickness = 5 mm, pixel spacing = 0.63 mm.

|  | train set (n=505) | | | validation set (n=104) | | | test set (n=104) | | |
| --- | --- | --- | --- | --- | --- | --- | --- | --- | --- |
|  | mean | median | range | mean | median | range | mean | median | range |
| repetition time [msec] | 2055 | 1807 | [1314; 4440] | 1996 | 1806 | [1313; 3449] | 2057 | 1810 | [1300; 3315] |
| echo time [msec] | 80 | 80 | [80; 136] | 81 | 80 | [80; 136] | 81 | 80 | [80; 136] |
| flip angle [°] | 90 | 90 | - | 90 | 90 | - | 90 | 90 | - |
| slice thickness [mm] | 4.94 | 5 | [4;5] | 4.97 | 5 | [4;5] | 4.97 | 5 | [4;5] |
| pixel spacing [mm] | 0.82 | 0.88 | [0.49; 1.23] | 0.83 | 0.88 | [0. 52; 0.88] | 0.82 | 0.88 | [0.56; 0.99] |
| matrix size | 481 | 432 | [320; 784] | 472 | 432 | [432; 704] | 482 | 432 | [384; 768] |
| field of view [mm] | 385 | 380 | [300; 490] | 386 | 380 | [330; 460] | 386 | 380 | [330; 440] |

**Table S2**: Mean, median, and range of basic sequence parameters of T2-weighted MRI sequences of the images used in the study, separated into training validation and test dataset

**S3 preprocessing**

In this study, the unsegmented single slice images were transformed to the median image size of the dataset of 432² by applying cubic interpolation. Subsequently, signal intensity were Z-Score normalized for the segmentation and the classification method, i.e. signal intensities are transformed to a mean of zero and a standard deviation (σ) of one. Prior to normalization, a body mask was defined by applying a threshold at signal intensity of 100 and only the image values within the mask were taken into account for normalization. This threshold was determined empirically. Image values outside 3σ were clipped [1].

The segmented images were cut to the smallest square image sections, which were large enough to completely contain the segmented liver. These images were interpolated to 234², which was the median image size of these sections of the entire dataset used for method development.

To improve the robustness of the segmentation and classification methods against overfitting, all images were augmented by affine transformations during training. Prior to augmentation the 234² sized images were first written centrally into a 256² matrix initialized with zero, in order not to scale and rotate image areas of the liver out of the matrix during augmentation. Then, with a probability of 75%, images were scaled by 0% to 5% and rotating by -10° to 10° along the z-axis while assembling the batches.

When applying Transfer Learning to images of a new subject, it is important to ensure that the images have similar image values to those of the images used for the original training. This can be achieved by matching the mean and standard deviation of the images to the values of the images of the original training [2].

Thus, the following steps were applied:

i) The image values in the interval [-$3\sigma3\sigma]$ were transformed to the interval [$0$ $1$]

ii) The images were copied into three channels to obtain the same image matrix as the 3-channel images of the ImageNet data set

iii) The mean of the channels were transformed to 0.485, 0.456, 0.406 and the standard deviation of the channels to 0.229, 0.224, 0.225 in order to match with the training data from ImageNet archive, according to pytorch’s documentation [3]

**S4 Model descriptions**

A ResNet50 architecture pre-trained on ImageNet with 50 trainable layers was used to classify the presence of cirrhotic alterations in the images [4].

For liver segmentation a ResNet34 architecture pre-trained on ImageNet with originally 34 trainable layers was applied. A decoder consisting of twelve convolution layers with Relu activation and Batch-Normalization replaced the last fully connected layer. After every second convolution the feature map size is up-scaled using sub-pixel convolution with pixel shuffle arrangement and connected to feature maps of the same size of the ResNet34 Encoder [5]. Thus a U-net similar architecture was used for the segmentation [6].

**S5 Experimental design**

All experiments were performed in pytorch and the application programming interface fastai [3,7]. The training was performed on a Nvidia Titan RTX Graphics Processing Unit (GPU) with 24 gigabyte video memory, using mixed precision training.

The training consisted of two phases with three sub-stages each, resulting in six training stages total, as presented in Table 3. In the first phase, the ResNet50 served as feature extractor, i.e. only the output layer was trained and all parameters of the pre-trained convolution layers were kept constant. In the second phase, the parameters of the pre-trained convolutional layers were made variable. The sub-stages consisted of 10 experiments that started from the model state with the highest validation accuracy of its previous sub-stages, but where the learning rate (LR) was modified, as detailed below. Early stopping was applied for implicit regularization [8]. Training was performed with the Adam optimization algorithm and the cross-entropy loss function [9]. A weight decay of 0.01 was implemented as described by Loshchilov et al. [10].

The LR and momentum followed a cyclical change during each training. Number of epochs was 80. In the first stage (training the output layer only), the LR was increased to a maximum value of 0.1 until 30% of the epochs were reached, starting from the maximum value divided by 25. Then the LR was dropped towards zero by cosine annealing until the last epoch. The momentum was dropped from 0.95 to 0.85 and then raised back to 0.95. The following stages were trained with the same cyclic change of the momentum and LR. However, only 40 epochs were trained each stage and the maximum LR was lowered to 0.01 in the second stage and 0.001 in the third stage.

In stage four, the pre-trained parameters of the convolutional layers were unfrozen. Additional 40 epochs were trained, however the maximum LR of the individual CNN layers were varied linearly from ${10}^{-6}$ to ${10}^{-4}$ (from the first to the output layer). In the fifth stage, additional 40 epochs were trained with a maximum LR that varied linearly from ${10}^{-5}$ to ${10}^{-4}$. In the last stage, again additional 40 epochs were trained with a maximum LR of ${10}^{-4}$for all layers of the CNN. The batch sizes used for training of the classification CNN were 256 for the segmented images and 136 for the unsegmented.

The four training stages of the segmentation network also followed the same scheme of a cyclic LR and momentum. In the first stage, the pre-trained parameters of the ResNet34 encoder were frozen and the number of epochs was 80. The LR was increased to a maximum value of 0.001 and then dropped to zero. In the second stage, the pre-trained parameters of the ResNet34 encoder were unfrozen. Additional 40 epochs were trained, however the maximum LR of the individual CNN layers were varied linearly from ${5*10}^{-6}$ to ${5*10}^{-4}$ (from the first to the output layer). In the third stage, additional 40 epochs were trained with a maximum LR that differed linearly from ${5*10}^{-5}$ to ${5*10}^{-4}$. Lastly, in the fourth stage, additional 40 epochs were trained with a maximum LR of ${5*10}^{-4}$for all layers of the CNN. While training the segmentation CNN a batch size of 40 was used.

**Supplement References**

1. Collewet G, Strzelecki M, Mariette F (2004) Influence of MRI acquisition protocols and image intensity normalization methods on texture classification. Magn Reson Imaging 22(1):81–91

2. Van Engelen A, van Dijk AC, Truijman MTB et al. (2015) Multi-center MRI carotid plaque component segmentation using feature normalization and transfer learning. IEEE Trans Med Imaging 34(6):1294–1305

3. Paszke A, Gross S, Massa F et al. (2019) PyTorch: An Imperative Style, High-Performance Deep Learning Library. Advances in Neural Information Processing Systems, pp. 8026-8037

4. He K, Zhang X, Ren S, Sun J (2016) Deep Residual Learning for Image Recognition. Proceedings of the IEEE conference on computer vision and pattern recognition, pp. 770-778

5. Shi W, Caballero J, Huszár F et al. (2016) Real-Time Single Image and Video Super-Resolution Using an Efficient Sub-Pixel Convolutional Neural Network. Proceedings of the IEEE conference on computer vision and pattern recognition, pp. 1874-1883

6. Ronneberger O, Fischer P, Brox T (2015) U-Net: Convolutional Networks for Biomedical Image Segmentation. International Conference on Medical image computing and computer-assisted intervention, Springer, Cham, pp. 234-241

7. Howard J, Gugger S (2020) Fastai: A Layered API for Deep Learning. Information 11(2):108

8. Zhang C, Bengio S, Hardt M, Recht B, Vinyals O (2016) Understanding deep learning requires rethinking generalization. arXiv preprint arXiv:1611.03530

9. Kingma DP, Ba J (2014) Adam: A Method for Stochastic Optimization. arXiv preprint arXiv:1412.6980

10. Loshchilov I, Hutter F (2017) Decoupled Weight Decay Regularization. arXiv preprint arXiv:1711.05101
